# Emerging Role of Lymphocyte Antigen-6 Family of Genes in Cancer and Immune Cells

**DOI:** 10.3389/fimmu.2019.00819

**Published:** 2019-04-24

**Authors:** Geeta Upadhyay

**Affiliations:** Department of Pathology, John P. Murtha Cancer Center, F. Edward Hebert School of Medicine, Uniformed Services University of the Health Sciences, Bethesda, MD, United States

**Keywords:** LY6D, LY6E, LY6H, LY6K, TGF-β, Immune, Oncology

## Abstract

Stem Cell Antigen-1 (*Sca-1/Ly6A*) was the first identified member of the Lymphocyte antigen-6 (*Ly6*) gene family. Sca-1 serves as a marker of cancer stem cells and tissue resident stem cells in mice. The Sca-1 gene is located on mouse chromosome 15. While a direct homolog of Sca-1 in humans is missing, human chromosome 8—the syntenic region to mouse chromosome 15—harbors several genes containing the characteristic domain known as LU domain. The function of the LU domain in human *LY6* gene family is not yet defined. The *LY6* gene family proteins are present on human chromosome 6, 8, 11, and 19. The most interesting of these genes are located on chromosome 8q24.3, a frequently amplified locus in human cancer. Human *LY6* genes represent novel biomarkers for poor cancer prognosis and are required for cancer progression in addition to playing an important role in immune escape. Although the mechanism associated with these phenotype is not yet clear, it is timely to review the current literature in order to address the critical need for future advancements in this field. This review will summarize recent findings which describe the role of human LY6 genes—*LY6D, LY6E, LY6H, LY6K, PSCA, LYPD2, SLURP1, GML, GPIHBP1*, and *LYNX1*; and their orthologs in mice at chromosome 15.

## Introduction

*Sca-1* is among the first identified members of the murine *Ly6* gene family (1, 2). The *Ly6* gene family belongs to the superfamily of lymphocyte antigen-6 (*Ly6*)/urokinase-type plasminogen activator receptor (uPAR) proteins. This superfamily is characterized by the presence of LU domain. LU domain is a 60–80 amino acid domain, which is composed of 6–10 cysteines arranged in a specific spacing pattern that allows distinct disulfide bridges which create the three-fingered (3F) structural motif. Three-fingered structural motif are ancient proteins. The LU domain, a three-fingered motif in Ly6/uPAR family are believed to be the evolutionary ancestors of 3FTx toxins found in snake venom. The LU domain in human LY6/uPAR family is not toxic, exact function of LU domain is not yet defined. LU domain is also found in extracellular domains of cell-surface receptors with membrane spanning domain (activin type 2 receptor and bone morphogenetic type IA receptor), or in GPI-anchored proteins CD177 or in secreted globular proteins such CD59 antigen, SLURP1/2.

The *LY6* gene family of proteins are located on human chromosomes 6, 8, 11, and 19 and the orthologs exist on syntenic areas of mouse chromosomes. The human chromosome 8 harbors genes namely *PSCA, LY6K, SLURP1, LYPD2, LYNX1/SLURP2, LY6D, GML, LY6E, LY6L, LY6H*, and *GPIHBP1*; while the syntenic mouse chromosome 15 contains genes *Psca, Slurp1, Lypd2, Slurp2, Lynx, Ly6d, Ly6g6g, Ly6k, Gml, Gml2, Ly6m, Ly6e, Ly6i, Ly6a, Ly6c1, Ly6c2, Ly6a2, Ly6g, Ly6g2, Ly6f*, *Ly6l, Ly6h*, and *Gpihbp1*. The human chromosome 19 harbors genes *LYPD4, CD177, TEX101, LYPD3, PINLYP, PLAUR, LYPD5*, and *SPACA4*; while the syntenic mouse chromosome 7 contains genes *Lypd5, Plaur, Pinlyp, Lypd3, Tex101, Lypd10, Lypd11, Cd177, Lypd4*, and *Spaca4*. The human chromosome 11 harbors genes *ACRV1, PATE1, PATE2, PATE3*, and *PATE4, CD59*; while the syntenic mouse chromosome 9 contains genes *Pate4, Pate2, Pate13, Pate3, Pate1, Pate10, Pate7, Pate6, Pate5, Pate12, Pate11, Pate9, Pate8, Pate14*, and *Acrv1*. The human chromosome 6 harbors genes *LY6G6C, LY6G6D, LY6G6F, LY6G5C*, and *LY6G5B* while the syntenic mouse chromosome 17 contains genes *Ly6g6c, Ly6g5c, Ly6g5b, Ly6g6d, Ly6g6f*, and *Ly6g6e* (2). Many of the mice Ly6 genes were lost in humans, perhaps due to their redundant function during evolution (3).

In this mini review, we will focus on the role of human LY6 gene family located on the chromosome 8 namely *LY6D, LY6E, LY6H, LY6K, PSCA, LYPD2, SLURP1, GML, GPIHBP1*, and *LYNX1* and their orthologs in mice at chromosome 15. We chose to focus on this set of genes as they have shown to be increased in human cancer. It is to note that the most widely studied murine Ly6 gene on chromosome 15, *Sca-1/Ly6A* does not have a human ortholog. It is not clear which gene in human Ly6 gene family may be functionally similar to *Sca-1* or the multiple genes may perform several important Sca-1 functions. Sca-1 protein is the most common cell surface marker that is used to enrich adult hematopoietic stem cells (HSCs). Sca-1 protein expression is variable depending on the stages of differentiation of HSCs. Sca-1 protein expression is reduced in HSCs that have differentiated to common myeloid progenitors and then re-expressed in subsets of myeloid progenitors. In a similar pattern, HSCs differentiating into progenitor population suppress Sca-1 expression and immature thymocytes have turned off the Sca-1 protein expression whereas mature single positive thymocytes and peripheral T- cells regain Sca-1 protein expression (4). Sca-1 protein expression has also been identified as an important regulator of tumor progression in mouse models of cancer (5–7). Although the role of LY6 genes on human chromosome 8 in immune cells is not yet established, their role in cancer progression is rapidly emerging. Therefore, we will discuss recent advances, current research gaps and critical need for future research regarding members of the LY6 gene family namely *LY6D, LY6E, LY6H, LY6K, PSCA, LYPD2, SLURP1, GML, GPIHBP1*, and *LYNX1*; and their orthologs in mice at chromosome 15.

### Lessons Learned From the Phenotype of Knockout Mice

The knockout mice models for mouse Ly6 genes with human orthologs—namely *Ly6E, Ly6K, Lynx1, Slurp1*, and *Gpihbp1* have been described (Table 1). The other family members have not been characterized in the knockout mice model. The phenotype and the characterization of the known knockout mice models are as described below, which offer important insight into the function of these genes.

**Table 1:** The phenotype of knockout mice model of Ly6 gene family members.

**Gene name**			
**Mice**	**Human ortholog**	**Knockout ^**(−/−)**^ mice, phenotype**	**Mechanism behind the phenotype**
Ly6E	Ly6E	Embryonic lethal due to placental defect	SynTA receptor pathway
Ly6K	Ly6K	Viable adult male (infertile) and female (fertile)	Unknown
Lynx1	Lynx1	Viable adult mice with no apparent phenotype	Not Applicable
Slurp1	Slurp1	Viable adult mice with palmoplantar keratoderma, metabolic and neuromuscular abnormal phenotype	Unknown
GPIHBP1	GPIHBP1	Viable adult mice with hypertriglyceridemia phenotype	Lipolysis pathway

*The mouse Ly6 genes namely Ly6E, Ly6K, Slurp1, Lynx1, and Gpihbp1 have mouse orthologs and they have knockout mice described. The mouse Ly6 genes namely Pcsa, Lypd2, Ly6D, Gml, and Ly6H have human orthologs but the knockout mice have not been described. The mouse Ly6 genes Sca1, Ly6I/M, Ly6C1, Ly6C2, Ly6B, Ly6G, BC025446, 9030619P08Rik don't have human orthologs. Among these genes only Sca1 has been described in a knockout mice model*.

***Ly6E:*** The mouse *Ly6E* gene was deleted by gene targeting in mice to create homozygous knockout mice (8). Animals with a *Ly6E*^−/−^ mutation showed embryonic lethality at E14.5. It was though that embryonic lethality was due to cardiac malformation. It was later resolved that *Ly6E* knockout induced lethality was due the critical role of *Ly6E* in the trophoblast stem cells (9). The trophoblast forms the outer layer of fetal part of the placenta. *Ly6E* is expressed specifically in the syncytiotrophoblast (SynT-I) cells (10). *Ly6E* was found to be a possible receptor for Syncytin A ligand (11). The syncytiotrophoblast layer of fetal placenta play important role in connecting with maternal placenta for the proper exchange of nutrients. In the absence of *Ly6E* in the fetal placenta, this fetal-maternal placental vascularization is not well-formed causing harm to the fetus. The role of *Ly6E* in human placental development has not yet been described. Thymic development was found to be normal in *Ly6E*^−/−^ mice, suggesting that *Ly6E* may play a redundant role in thymic development and development of immune cells.

***Ly6K:*** The mouse *Ly6K* gene knockout mice were generated using a targeting vector substituting exons 2, 3, and 4 (12). Adult *Ly6K*^−/−^ mutant male mice were found to be infertile. Female *Ly6K*^−/−^ mutant mice had normal fertility. *Ly6K* is not expressed in mature spermatozoa, however the spermatozoa from *Ly6K*^−/−^ mutant male mice were unable to migrate into the oviduct (12). The reason for *Ly6K* associated migration defects in sperm of adult mice is not yet known. The role of *Ly6K* in human sperm related infertility is not yet described.

***Slurp1:*** The mouse *Slurp1* gene is a secreted member of the *Ly6* gene family. The deficiency and mutations in human *SLURP1* gene causes Mal de Meleda (MDM), a rare autosomal recessive genetic disease, characterized by inflammatory palmoplantar keratoderma (13, 14). *Slurp1*^−/−^ mutant mice exhibit this rare palmoplantar keratoderma, and show metabolic phenotypes such as reduced adiposity, protection from obesity on a high-fat diet, low plasma lipid levels, and neuromuscular abnormalities such as hind-limb clasping (15).

***Gpihbp1*:** Glycosylphosphatidylinositol-anchored high-density lipoprotein-binding protein 1 (*Gpihbp1*) deficient mice on a regular chow diet display accumulation of chylomicrons in the plasma and high plasma triglyceride levels (16). *Gpihbp1*^−/−^ mice show reciprocal metabolic perturbations in adipose tissue and liver due to defective lipolysis (17). These results suggest that human GPIHBP1 gene may play important role in the lipolytic processing of triglyceride-rich lipoproteins.

***Lynx1:*** Ly6 neurotoxin1 (*Lynx1*) knockout mice display increased visual cortex plasticity in mice (18). The *Lynx1*^−/−^ phenotype was partially derived from the inhibition of nicotinic acetylcholine receptor signaling by *Lynx1* (18). These results suggest that human LYNX1 gene may play important role in nicotine response in the brain.

### Expression of Mouse Ly6 Genes in Immune Cells

The expression of many mouse *Ly6* gene family proteins are lineage specific and their expression coincides with the differentiation stages of leukocyte cell populations as seen in the mouse model. The role of human LY6 genes in immune cells differentiation is still not clear. These properties have made them attractive targets to be used for subset identification of leukocytes *in vitro* and antibody-mediated depletion of specific immune cell populations *in vivo*. Recently, Nigrovic et al. summarized the mRNA expression of mice *Ly6* genes in B-cells, T-cells, NK cells, monocytes, and dendritic cells based on data from the Immgen database (3). *Ly6E* RNA expression was expressed on all tested immune cell subtypes in the Immgen database. *Ly6D* RNA was expressed in B-cells, T-cells and dendritic cells. Lypd2, which was described in non-classical monocytes (19). *Ly6F, Ly6H, Ly6K, Gml, Psca, Gpihbp1* RNA expression was not found to be associated with expression on immune cells in mice. Interestingly, the mice genes namely *Sca1, Ly6B, Ly6C, Ly6G, Ly6I/Ly6M*, and *Ly6F* RNA expression have found to be expressed on many immune cells types in mice. These genes do not have human orthologs. It is yet to be discovered which human genes may function similar to these mice genes. The mRNA expression for *Ly6A/Sca1* was found to be present in B-cells, T-cells and dendritic cells (3). *Ly6B* RNA expression was found in NK cells, monocytes and neutrophils but absent from B-cells, T-cells and dendritic cells. *Ly6C* RNA expression was found to be present in all subsets except T cells. *Ly6G* RNA expression was only present in neutrophils and absent from all other cell types analyzed. *Ly6I/Ly6M* RNA expression was present only in monocytes and neutrophils and absent in all other cell types. It has been shown that RNA and protein expression for some of the human LY6 gene family members such as GML (20, 21), *LY6K* (22, 23), PSCA (24, 25), and LY6E (23, 26) have immune-modulatory properties in tumor microenvironment.

*LY6E* and *LY6D* are the only two genes on human chromosome 8 with a mouse ortholog on chromosome 15, for which some data is available to describe their expression and function in immune cells using mouse models, as discussed below. These genes have been shown to be important in the proliferation and differentiation of immune cells using mainly *in vitro* methods (3, 27). The mechanistic basis behind the differentiation specific expression remains to be fully understood.

***Ly6D*:** The mRNA for mouse *Ly6D* gene was found to be present in B-cells, T-cells and dendritic cells and absent in NK cells, monocytes, and neutrophils (3). Recent *in vitro* studies indicate that *Ly6D* gene is expressed in common lymphoid progenitors which arise from hematopoietic stem cells and give rise to B-lineage lymphocytes (28). Mouse *Ly6D* gene expression is associated with B-cell specification (29). *Ly6D* and *SiglecH* gene expression positive cells from IL7R positive lymphoid progenitor cell populations were committed to become plasmacytoid dendritic cells, while double negative cells were uncommitted. Plasmacytoid dendritic cells are an immune subset devoted to the production of high amounts of type 1 interferon in response to viral infections (30).

***Ly6E*:** The mouse *Ly6E* gene is expressed in peripheral B-cells, immature T-cells, activated T cells, thymus stromal cells, and macrophages (3). A functional role of *Ly6E* was reported in maintenance of self-renewal of erythroid progenitors (31).

### Expression of Human LY6 Genes in Normal (Non-lymphoid) Tissues

**LY6D:** Human LY6D RNA and protein is expressed in normal esophageal and skin as published by the human protein atlas on www.proteinatlas.org (32–36).

**LY6E:** Human *LY6E* RNA is expressed in normal liver, fetal placenta, lung, and spleen (32–36).

**LY6H:** Human *LY6H* RNA is expressed in brain (32–36).

**LY6K:** Human *LY6K* RNA and protein is expressed in normal testis (32–36).

**PSCA:** Human *PSCA* RNA is expressed in prostate, urinary bladder, and esophagus (32–36).

**LYPD2:** Human *LYPD2* RNA is expressed in esophagus and tonsil (32–36).

**SLURP1:** Human *SLURP1* RNA is expressed in esophagus and skin (32–36).

**GML:** Human *GML* RNA is expressed in testis and adrenal gland (32–36).

**GPIHBP1:** Human *GPIHBP1* RNA is expressed in adipose, lung, breast, brain, heart and soft (32–36).

### Expression of Human LY6 Genes Is Associated With Cancer and Outcome of the Disease

The *LY6D, LY6E, Ly6K*, and *Ly6H* RNA is expressed at high levels compared to adjacent normal tissues in a multitude of tumors including ovarian, colorectal, gastric, breast, lung, bladder, brain and CNS, cervical, esophageal, head and neck, and pancreatic cancer. The increased expression of *LY6D, LY6E, LY6K*, and *LY6H* was associated with poor survival in ovarian, colorectal, gastric, breast, and lung cancer (37) (Table 2). The casual reason for the increased expression of LY6 family proteins and the poor survival outcome is not yet established. As discussed further in the review under cell signaling heading, LY6 proteins play important role in TGFβ signaling, AKT pathways and immune regulation. A cumulative effect of Ly6 downstream pathways may lead to increased aggressiveness of cancer cells and leading to poor survival outcome. More mechanistic studies will need to be performed to determine the precise pathways responsible for poor survival outcome in patients.

**Table 2:** Correlation of high mRNA expression and patient survival outcome in multiple cancer types.

**Cancer type**	**Genes**	**Expression in tumors (*p* < 0.05)**	**Survival analysis (*p* < 0.05)**
Ovarian	LY6D	Up	Poor prognosis
	LY6E	Up	Poor prognosis
	LY6H	Up	Poor prognosis
	LY6K	Up	Poor prognosis
Colorectal	LY6D	Up	Poor prognosis
	LY6E	Up	Poor prognosis
	LY6H	Up	Poor prognosis
	LY6K	Up	Poor prognosis
Gastric	LY6D	Up	Poor prognosis
	LY6E	Up	Poor prognosis
	LY6H	Up	Poor prognosis
	LY6K	Up	Poor prognosis
Breast	LY6D	Up	Poor prognosis
	LY6E	Up	Poor prognosis
	LY6H	Up	Poor prognosis
	LY6K	Up	Poor prognosis
Lung	LY6D	Up	Poor prognosis
	LY6E	Up	Poor prognosis
	LY6H	Up	OS (NS), Others (NA)
	LY6K	Up	Poor prognosis
Bladder	LY6D	Up	OS (NS), Others (NA)
	LY6E	Up	Poor prognosis
	LY6H	NS	OS (NS), Others (NA)
	LY6K	Up	Poor prognosis
Brain and CNS	LY6D	Up	OS (NS), Others (NA)
	LY6E	Up	Poor prognosis
	LY6H	Up	OS (NS), Others (NA)
	LY6K	Up	Poor prognosis
Cervical	LY6D	Up	OS (NA), RFS (NS)
	LY6E	Up	OS (NA), RFS (NS)
	LY6H	Up	OS (NA), RFS (NS)
	LY6K	Up	OS (NA), RFS (NS)
Esophageal	LY6D	Up	OS (NS), Others (NA)
	LY6E	Up	OS (NS), Others (NA)
	LY6H	Up	OS (NS), Others (NA)
	LY6K	Up	OS (NS), Others (NA)
Head and neck	LY6D	Up	OS (NS), Others (NA)
	LY6E	Up	OS (NS), Others (NA)
	LY6H	Up	OS (NS), Others (NA)
	LY6K	Up	OS (NS), Others (NA)
Pancreatic	LY6D	Up	OS (NS), Others (NA)
	LY6E	Up	OS (NS), Others (NA)
	LY6H	Up	OS (NS), Others (NA)
	LY6K	Up	OS (NS), Others (NA)

**Figure 1:**
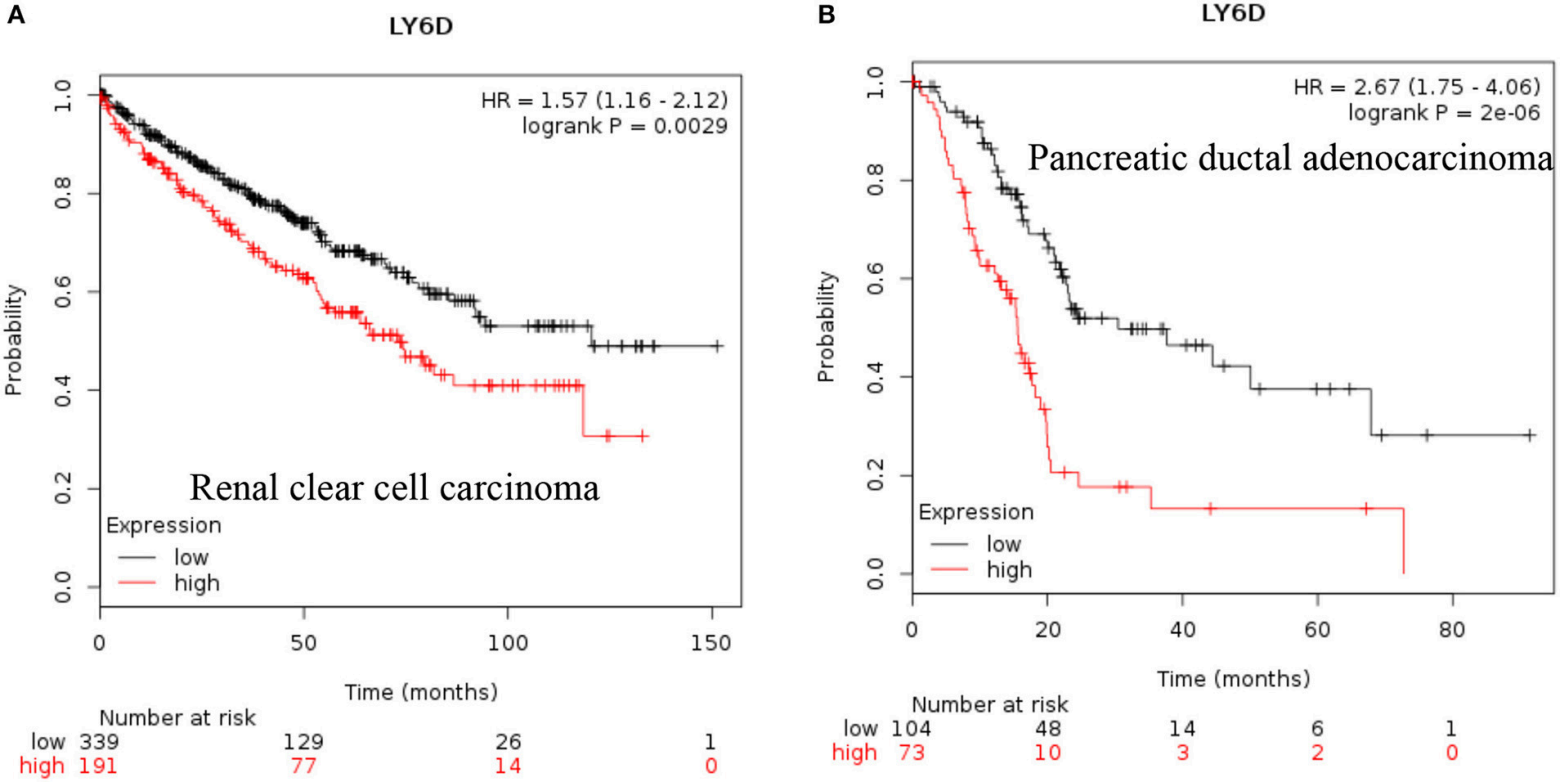
Increased LY6D mRNA expression in cancer and patient survival. High LY6D RNA expression leads to poor survival in **(A)** renal clear cell carcinoma, **(B)** pancreatic ductal adenocarcinoma. These data were recently added in Km plotter webtool from RNA seq pan cancer analysis (38).

**LY6D**: Recent clinical outcome data added and published by Km plotter web tool show that increased RNA expression of *LY6D* is associated with poor prognosis in renal clear cell carcinoma and pancreatic ductal adenocarcinoma [Figure 1, (38)]. Recently, *LY6D* expression—in addition to *OLFM4* and *S100A7*—was found to be associated with distant metastasis of estrogen receptor positive breast cancer (39). *LY6D* has also been shown to be increased in aggressive forms of head and neck cancer (40).

**Figure 2:**
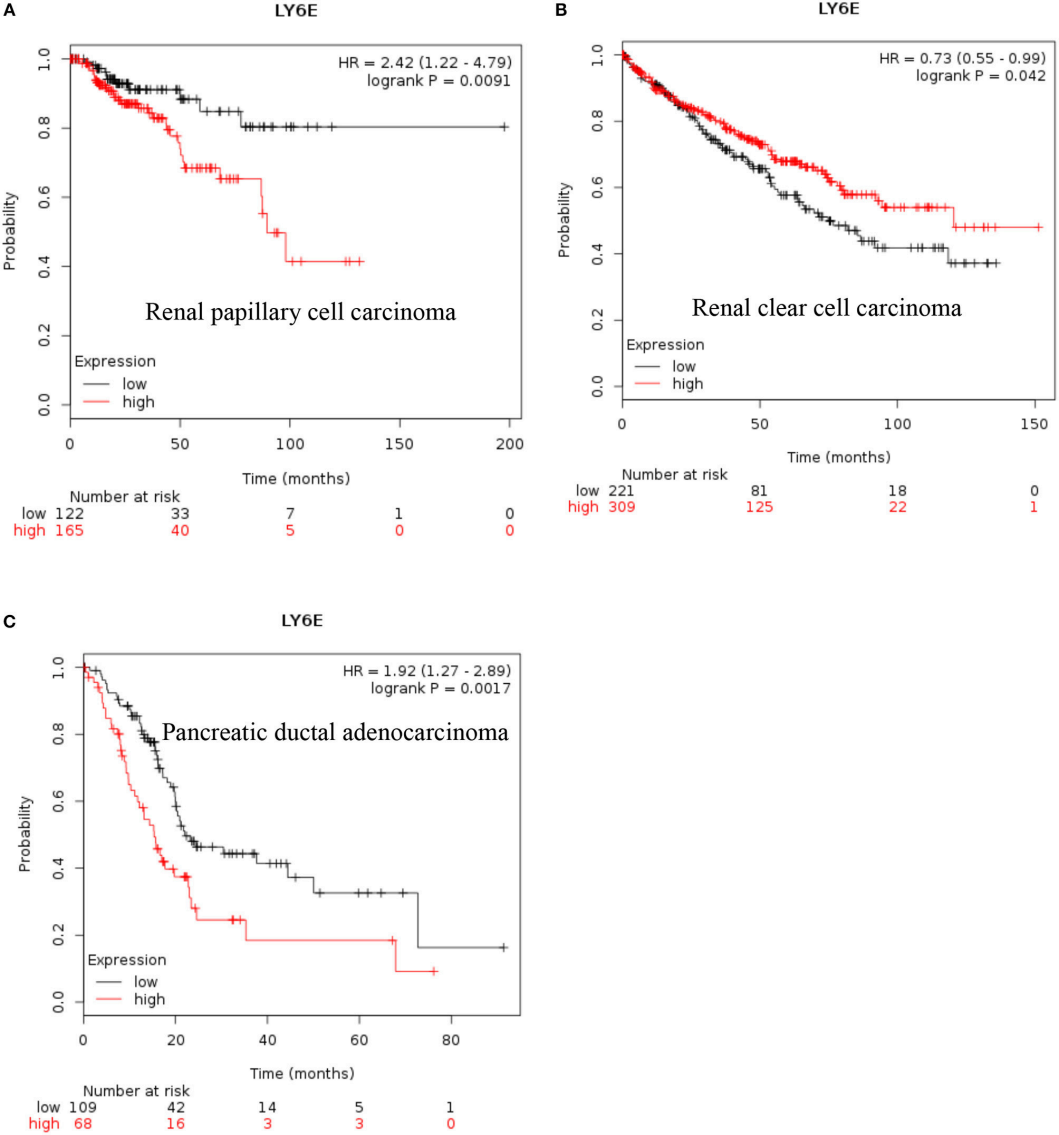
Increased LY6E mRNA expression in cancer and patient survival. High LY6E RNA expression leads to **(A)** poor survival in renal papillary cell carcinoma, **(B)** good prognosis for renal clear cell carcinoma, **(C)** poor survival in pancreatic ductal adenocarcinoma. These data were recently added in Km plotter webtool from RNA seq pan cancer analysis (38).

**LY6E:** Recent data also show that increased expression of *LY6E* is associated with poor overall survival of renal papillary cell carcinoma and is a good prognostic marker for renal clear cell carcinoma [Figures 2A,B, (38)]. These new data indicated that increased expression of *LY6E* is associated with poor overall survival of pancreatic ductal adenocarcinoma [Figure 2C, (38)]. The use of genome wide data analysis has prompted several new reports showing increased expression of *Ly6E* in bladder cancer, gastric cancer (39, 40). The *LY6E* gene has been also associated with more aggressive stem like cells in hepatocellular carcinoma, pancreatic carcinoma, colon, and kidney (41–43).

**Figure 3:**
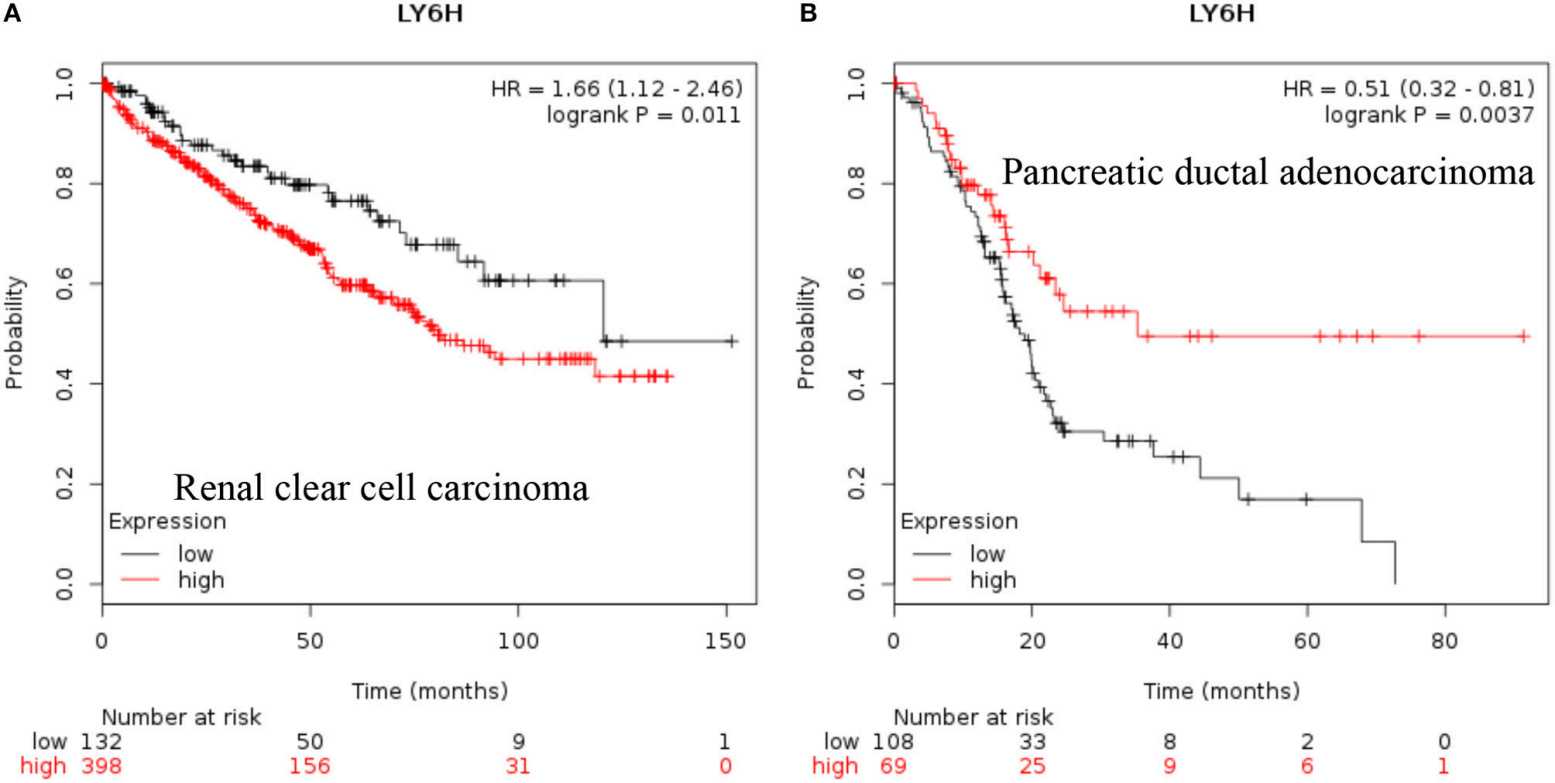
Increased LY6H mRNA expression in cancer and patient survival. High LY6H RNA expression leads to poor survival in **(A)** renal clear cell carcinoma, **(B)** pancreatic ductal adenocarcinoma (38).

**LY6H:** Recent data shows that increased expression of *Ly6H* is associated with poor overall survival of renal clear cell carcinoma and pancreatic ductal adenocarcinoma [Figure 3, (38)].

**Figure 4:**
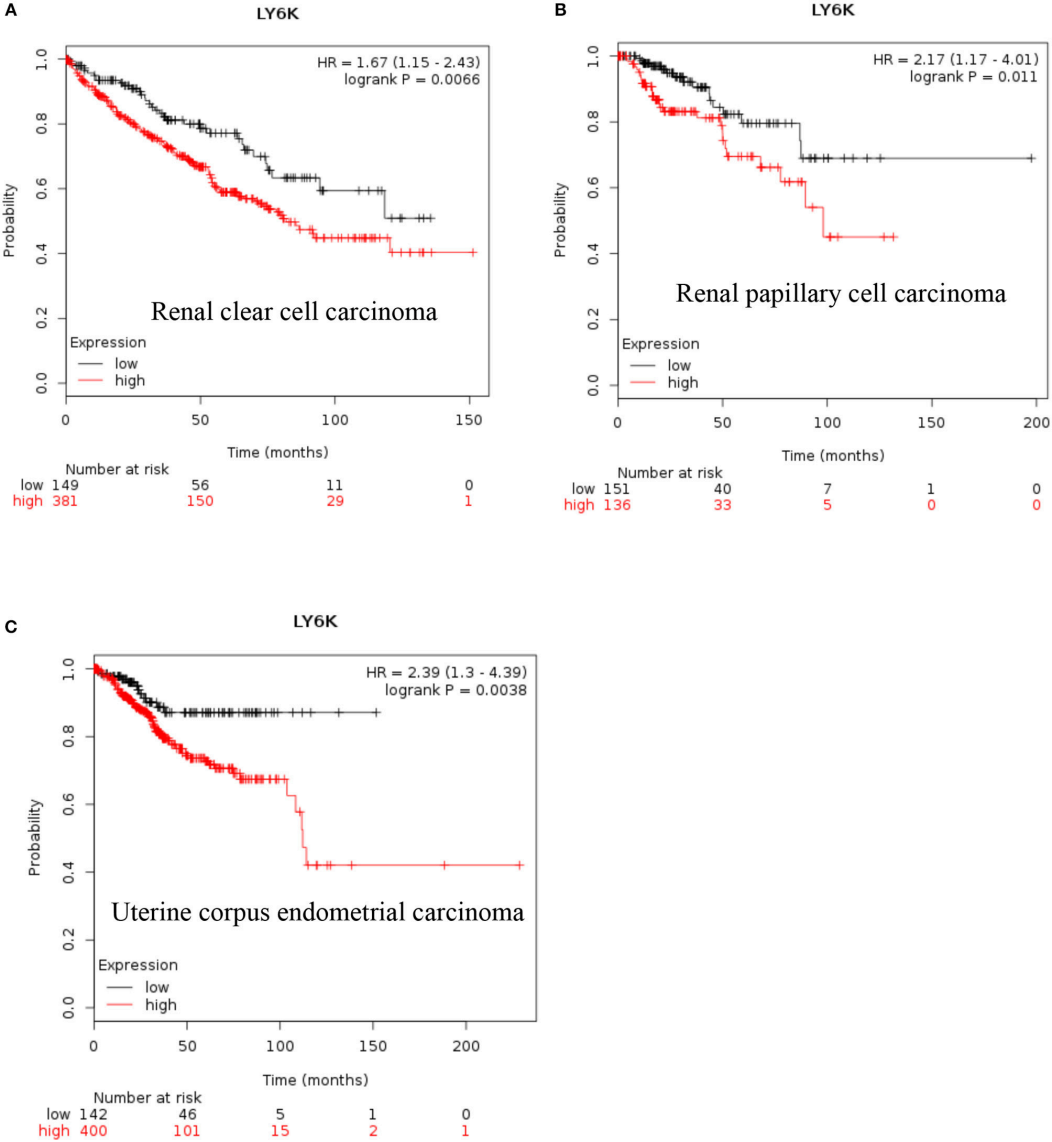
Increased LY6K mRNA expression in cancer and patient survival. High Ly6K RNA expression leads to poor survival in **(A)** renal clear cell carcinoma, **(B)** renal papillary cell carcinoma and, **(C)** pancreatic ductal adenocarcinoma (38).

**LY6K:** Increased expression of *LY6K* has also been reported in metastatic ER positive breast cancer (41–43), esophageal squamous cancer (44), gingivobuccal cancers (45), bladder cancer (46), and lung cancer (47). Recent data also show that increased expression of LY6K is associated with poor overall survival of renal clear cell carcinoma, renal papillary cell carcinoma and uterine corpus endometrial carcinoma [Figure 4, (38)].

**PSCA:** Prostate stem cell antigen (*PSCA*) RNA is expressed at high levels compared to adjacent normal tissues in prostate and pancreatic tumors as well as in glioma (48, 49). *PSCA* is downregulated in esophageal squamous cell carcinoma where it can act as a tumor suppressor by facilitating the nuclear translocation of RB1CC1 (50). Bioinformatics studies have indicated that polymorphisms rs2294008 in the *PSCA* gene may be prognostic in nature, however this association remains inconclusive due to contradictory observation and a lack of *in vitro* or *in vivo* experimental evidence (51–53).

**LYPD2:** *LYPD2* RNA is expressed at high levels compared to adjacent normal tissues in cervical and head and neck cancer are associated with a favorable prognosis as shown by the human protein atlas (32).

**SLURP1:** *SLURP1* RNA expression is reduced in metastatic melanoma (13). The prognostic value of Slurp1 is unknown.

**GML:** *GML* RNA expression is increased in non-small cell lung carcinoma (NSCLC) expressing wild-type P53 or P53 negative tumors. This specific expression of GML in P53 negative tumors was found to be linked to cisplatin sensitivity in NSCLC (54). The prognostic value of GML is not clear.

**GPIHBP1:** *GPIHBP1* RNA and protein is increased in renal cancer (32–35). The prognostic value of *GPIHBP1* is unknown.

### Role of Human LY6 Genes in Other (Non-cancer) Diseases

**LY6D:** A single nucleotide polymorphism rs2572886 found in the human *LY6D* and *Ly6PD2* genes is associated with cellular infection susceptibility to HIV-1 in lymphoblastoid B-cells and in primary T-cells and was also associated with accelerated disease progression in one of two cohorts of HIV-1–infected patients (55).

**LY6E:** *LY6E* expression is up-regulated during chronic HIV infection (26, 56). Using a HIV pathogenesis model, it was shown that *LY6E* can down-regulate monocyte responsiveness by modulating CD14 expression via an unknown mechanism (26). Recently, LY6E was shown to enhance viral infectivity IFN dependent fashion (57).

**GPIHBP1:** GPIHBP1 is an endothelial cell specific protein. It facilitates triglyceride (TG) lipolysis in the endothelial cells surface by binding to lipoprotein lipase (LPL), which releases nutrients to surrounding tissues (58). Four missense mutations in highly conserved residues in *GPIHBP1* (C65Y, C65S, C68G, and Q115P) gene were identified in severe chylomicronemia leading to buildup of triglyceride causing health issues such as pancreatitis (59).

### Cell Signaling Pathways Associated With Human LY6 Genes

The LY6 proteins are glycosylphosphatidylinositol (GPI)-anchored cell surface proteins, the regulation of LY6 RNA and protein is not yet understood very well.

#### TGF-β Signaling

Molecular analysis showed that LY6E and LY6K contribute to tumorigenic progression by increased TGF-β signaling, immune escape and increased INF-γ signaling (23).

#### PI3K Signaling

The PTEN and PI3K/Akt signaling pathways are involved in LY6E-mediated increase in HIF-1α transcription (60).

#### Nicotinic Acetylcholine Receptor Signaling

LYNX1, SLURP1, PSCA, and LY6H can modulate α7 nicotinic acetylcholine receptor (nAChR) signaling. The α7 nicotinic acetylcholine receptor (nAChR) protein is expressed in the central and peripheral nervous system (CNS), muscle, lung, and placenta. The nAChR signaling is associated with nicotine addiction, cancers of lung and liver, and preeclampsia (61, 62). In the brain, activation of α7nAChR in macrophages inhibits production of inflammatory cytokines (63). In colorectal cancer, the activation of α7nAChR in tumor macrophages inhibits colorectal cancer metastasis through the JAK2/STAT3 signaling pathway (64). α7nAChR has been considered an important drug target for the inhibition of lung cancer (65). On a cautionary note, α7nAChR is such an important neurotransmitter receptor in the CNS and muscle, that targeting nicotinic signaling directly via α7nAChR may cause multiple unwanted side effects. Nicotinic signaling can be modulated by multiple members of the LY6 gene family including LYNX1/2, SLURP1/2, and PSCA (66). Nicotinic signaling can affect glutamatergic signaling in in the hippocampus which may impact learning and memory. One member of LY6 family, LY6H have shown to play important role in glutamatergic signaling in the brain (67). It is yet to be determined if LY6H or other members of LY6 gene family regulate cross talk of nicotinic signaling and glutamergic signaling in the brain.

## Concluding Remarks

**Figure 5:**
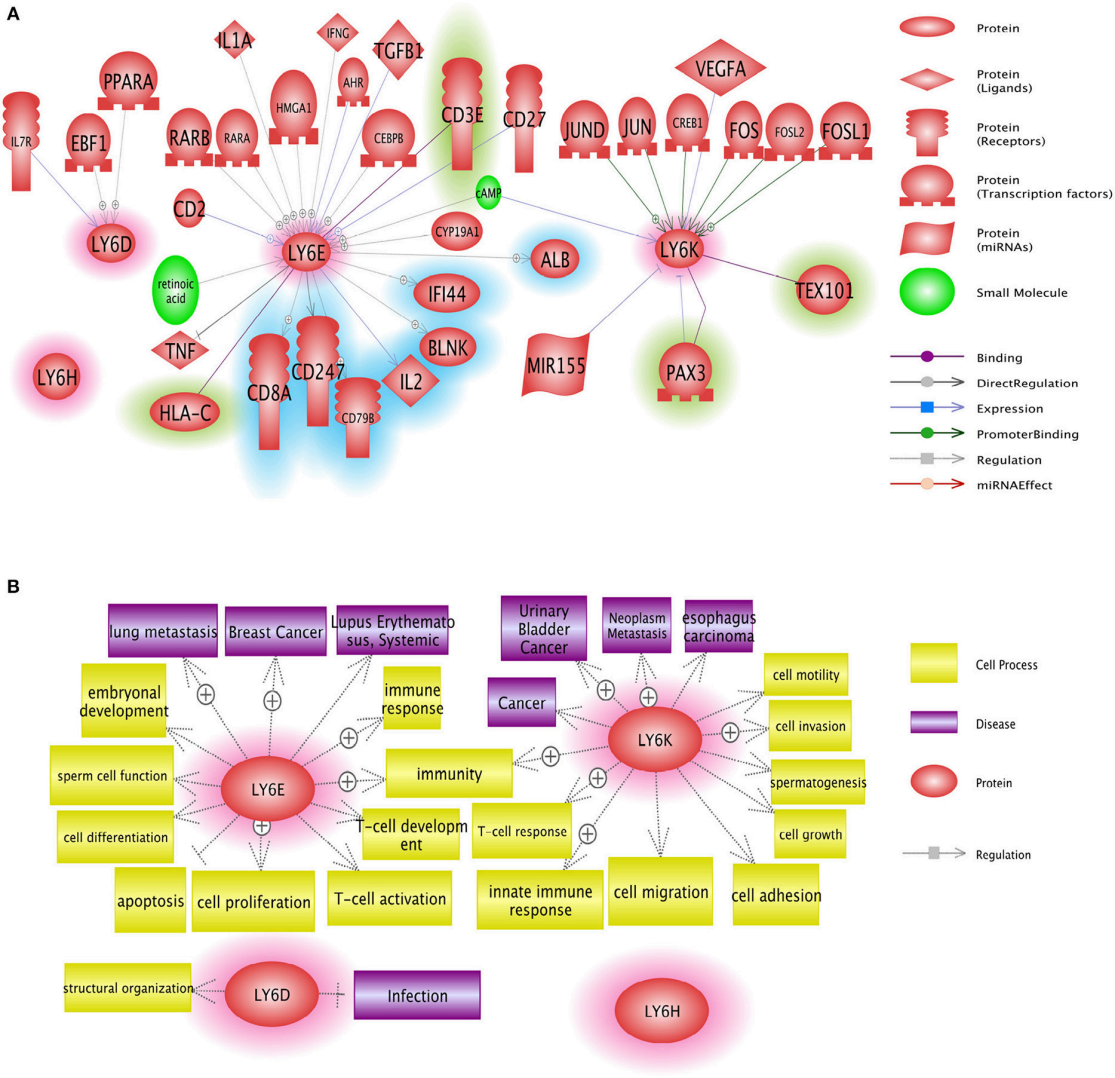
Network analysis of Ly6D, Ly6E, Ly6K, and Ly6H genes. **(A)** Pathway studio network analysis showed that LY6 signaling is involved in broad range of molecules including growth factor, nuclear receptor, and micro RNAs. The upstream regulators are not highlighted, the downstream effectors are highlighted with blue, and the potential binding partners are highlighted with green. **(B)** Pathway studio network analysis showed that Ly6 gene family affect multitude of cellular fate and cell-cell interaction with microenvironment ranging from growth, apoptosis, autophagy, immune response (37).

LY6 gene family members have the potential to be used as therapeutic targets. Several approaches including small molecules and antibody neutralization may be suitable in targeting LY6 family proteins. Ly6E protein expression was identified as a highly promising target for molecular antibody drug conjugates directed to solid tumors in animal models which show high RNA expression of LY6E (68). LY6D, LY6E, LY6H, and LY6K may be used in targeted cancer therapy, provided that future in-depth mechanistic studies reveal the signaling networks of these proteins in physiology and disease [Figure 5, (37)]. GPIHBP1 mimicking small molecules will have the potential to treat defective lipolysis of triglyceride due to mutated GPIHBP1. In depth analysis of regulation of LY6 gene expression and identification of downstream targets of LY6 proteins are required to better understand LY6 biology. The α7 nicotinic acetylcholine receptor (nAChR) signaling is at the center of a number of diseases including schizophrenia, Alzheimer's disease, chronic pain and inflammatory diseases (69). Therefore, the LY6 gene family members such as LYNX1, SLURP1, PSCA, and LY6H which modulate α7 nicotinic acetylcholine receptor (nAChR) signaling may be considered for the tissue-specific modulation of the nAChR pathway.

## Author Contributions

The author confirms being the sole contributor of this work and has approved it for publication.

### Conflict of Interest Statement

The author declares that the research was conducted in the absence of any commercial or financial relationships that could be construed as a potential conflict of interest.
